# Induction of Autophagy by a Novel Small Molecule Improves Aβ Pathology and Ameliorates Cognitive Deficits

**DOI:** 10.1371/journal.pone.0065367

**Published:** 2013-06-04

**Authors:** Cheng Chu, Xinjiang Zhang, Wei Ma, Li Li, Wei Wang, Lu Shang, Peng Fu

**Affiliations:** 1 Department of Neurology, The First People’s Hospital of Yangzhou, Jiang Su, PR China; 2 Department of Neurology, Changzheng Hospital, Second Military Medical University, Shanghai, PR China; 3 Mu Danjiang Medical College, Hei Longjiang, PR China; 4 The Eighth Detachment Health Team of CAPF, Shanghai Corps, Jiangsu, PR China; 5 Department of Pharmacy, Changhai Hospital,Second Military Medical University, Shanghai, PR China; University of Florida, United States of America

## Abstract

Growing evidence has demonstrated a neuroprotective role of autophagy in Alzheimer’s disease (AD). Thus, autophagy has been regarded as a potential therapeutic target, attracting increasing interest in pharmaceutical autophagy modulation by small molecules. We designed a two-cycle screening strategy on the basis of imaging high-throughout screening (HTS) and cellular toxicity assay, and have identified a novel autophagy inducer known as GTM-1. We further showed that GTM-1 exhibits dual activities, such as autophagy induction and antagonism against Aβ-oligomer toxicity. GTM-1 modulates autophagy in an Akt-independent and mTOR-independent manner. In addition, we demonstrated that GTM-1 enhances autophagy clearance and reverses the downregulation of autophagy flux by thapsigargin and asparagine. Furthermore, administration of GTM-1 attenuated Aβ pathology and ameliorated cognitive deficits in AD mice.

## Introduction

Alzheimer’s disease is one of the most common neurodegenerative diseases in the elderly and is characterized by progressive dementia and brain morphological illness, in which the brain becomes littered with the accumulation of fibrillogenic amyloid-β peptide (Aβ) oligomers [1]–[6]. Aβ is a peptide that is 40 or 42 amino acid residues in length (Aβ40 and Aβ42) and that is predominantly derived from the amyloid precursor protein (APP) upon its sequential cleavage by BACE1 and the γ-secretase complex [3], [7]. Thus, when a potential therapeutic agent is assessed in pre-clinical studies using cellular or mouse models, it is imperative to consider its concomitant effects on Aβ. Between the two predominant forms of Aβ, Aβ40 and Aβ42, Aβ42 demonstrates a greater propensity to self-aggregate into insoluble fibrils compared to Aβ40 [8]–[10]. Thus, Aβ42 demonstrates a more enhanced toxicity in neuronal diseases and is often adopted in the generation of neurodegenerative cellular models.

Overwhelming evidence has highlighted the role of autophagy in several neurodegenerative diseases including AD [11]–[14]. Indeed, it has been well documented that a decrease in autophagy function might contribute to the accumulation of protein in the brain [3], [5], [14], [15], [16]. Moreover, both cellular and animal studies have shown that autophagy is neuroprotective against the accumulation of cytotoxic proteins [4], [5], [8], [11], [12], [13].

Macroautophagy, which will herein be referred to as autophagy, is a lysosome-dependent cellular catabolic mechanism mediating the turnover of dysfunctional organelles, aggregated proteins and involves the sequestration of material inside double-membrane vesicles known as autophagosomes. In eukaryotes, the core machinery of autophagy includes the following three important steps: (1) induction, which is initiated by activation of the Atg1 complex. Atg1 and Atg13 are two key components (and others) in this complex, and mTOR inhibits activation of the Atg1 complex by super-phosphorylation of Atg13, which subsequently results in inhibition of autophagy induction [17], [18]; (2) vesicle nucleation and expansion, which is the initial step that recruits proteins and lipids for autophagosome construction and which requires activation of the Beclin1 and Vps34 (phosphatidylinositol 3-kinase) complex; (3) autolysosome formation, which occurs when the autophagosome is completed, and subsequently fuses with the lysosome to form an autolysosome vesicle, in which the cytosolic cargos will be degraded. The resulting products will then be released back into the cytosol for recycling [17], [18].

Growing evidence has demonstrated a neuroprotective role of autophagy in mediating the degradation of aggregated proteins that cause AD. Thus, autophagy is regarded as a potential therapeutic target to decrease detrimental Aβ aggregates in neurons and alleviate neurotoxicity [2], [4], [5], [11], [13], [15]. Several studies have focused on pharmaceutical autophagy modulation using known autophagy inducers or novel small molecules that have been identified from cell-based screening [4], [5], [19]–[22].

In recent years, several important studies have reported small molecules with a quinazoline structure, which demonstrate a potential ability to modulate cellular autophagy, such as SMER28 and Spautin-1 [23]–[25]. Our group obtained a compound library (120 compounds) with various phthalazinone structures in order to identify small molecules that can alleviate neurodegenerative diseases, particularly, Alzheimer’s disease (AD), via cellular autophagy regulation.

In this study, we designed a two-cycle screening strategy on the basis of an imaging HCS strategy and cellular toxicity assay, and identified a novel autophagy inducer GTM-1 from the compound library. We showed that GTM-1 demonstrates dual activities: autophagy induction and antagonism against Aβ-oligomer toxicity; moreover, it can rapidly and efficiently induce autophagy in neurons. In addition, we also showed that GTM-1 modulates autophagy in an Akt-independent and mTOR-independent manner, which differs from the effects of rapamycin. Importantly, GTM-1 also had the ability to reverse the inhibition of the autophagy flux induced by thapsigargin and asparagine, which conferred neuroprotection. Moreover, GTM-1-mediated the upregulation of autophagy, which attenuated Aβ pathology and ameliorated cognitive deficits observed in Alzheimer’s disease mice. Taken together, these findings indicated that GTM-1 may represent a potentially effective target compound for AD therapeutic drug treatment.

## Results

### A Novel Small Molecule Stimulates Autophagy in Neurons and Attenuates Aβ Oligomer-induced Neurotoxicity

In recent years, several interesting reports have demonstrated that compounds with a quinazoline scaffold play an important role in the regulation of cell autophagy, such as SMER28 and Spautin-1 [23]–[25]. Our lab obtained a compound library consisting of phthalazinone derivatives (120 compounds).

SH-SY5Y, a human derived neuroblastoma cell line, has been widely used in the study of many neurodegenerative diseases, including AD [27]. We established an SH-SY5Y/LC3-GFP cell line that stably expressed human microtubule-associated protein (MAP) LC3-GFP. Previous reports have indicated that LC3-GFP specifically labels autophagosomal membranes, and thus, LC3-GFP spots may be used to quantitatively analyze autophagosomes [28]. Using SH-SY5Y/LC3-GFP cells, we identified 15 inducers of autophagy (data not shown) from the phthalazinone compound library using an imaging high-content screening strategy [25].

**Figure 1:**
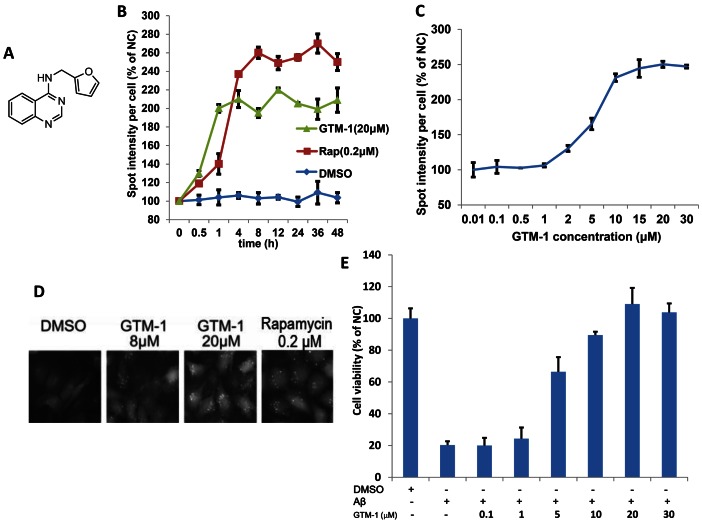
Isolation of a small-molecule inducer of autophagy in neurons. A, The structure of GTM-1. B–D, GTM-1 increases the spot intensity of LC3-GFP+ puncta. SH-SY5Y/LC3-GFP cells were treated with 20 µM GTM-1 for the indicated times (B) or with the indicated concentrations (C) for 24 hrs. The spot intensity of LC3-GFP+ puncta in SH-SY5Y/LC3-GFP cells treated with control vehicle at first time point (0 hr) was used as the negative control (NC). The image data were expressed as % of NC. For each treatment condition, 1000 cells were analyzed. The cells were imaged using a fluorescence microscope (D). E, SH-SY5Y cells were incubated with the indicated compounds for 24 hrs. The cell viability was assayed using the MTT assay. Aβ, Aβ42 (30 µM). Cells treated with vehicle (0.1% DMSO) were used as a negative control (NC).

A previous report conducted by Hung et al. adopted extracellular Aβ to mimic the brain morphological damages caused by fibrillogenic Aβ at the cellular level. It has been demonstrated that extracellular Aβ can be easily internalized, causing neuronal apoptosis after simple incubation [29]. On the basis of this finding, we performed an Aβ-induced cytotoxicity test. From this study, 15 hits were further examined using the cellular Aβ-induced cytotoxicity test [29] in the second screening (studies examining the roles of these other hits are still ongoing). Using this assay to examine the extent of neuroprotection offered by these compounds, we identified a quinazoline derivative, GTM-1 (Fig. 1A), which exhibited dual activities in autophagy induction and antagonism against Aβ-oligomer toxicity (Fig. 1E). The NMR spectrum of GTM-1 is shown in figure 1 (Fig. 1). A quantitative analysis of the LC3-GFP puncta induced by GTM-1 (Fig. 1C) using HCS demonstrated that the autophagy induction of GTM-1 in SH-SY5Y/LC3-GFP cells was as rapid as 4 hrs (Fig. 1B) and occurred in a dose-dependent manner with an EC_50_ of 7.9 µM (Fig. 1C).

**Figure 2:**
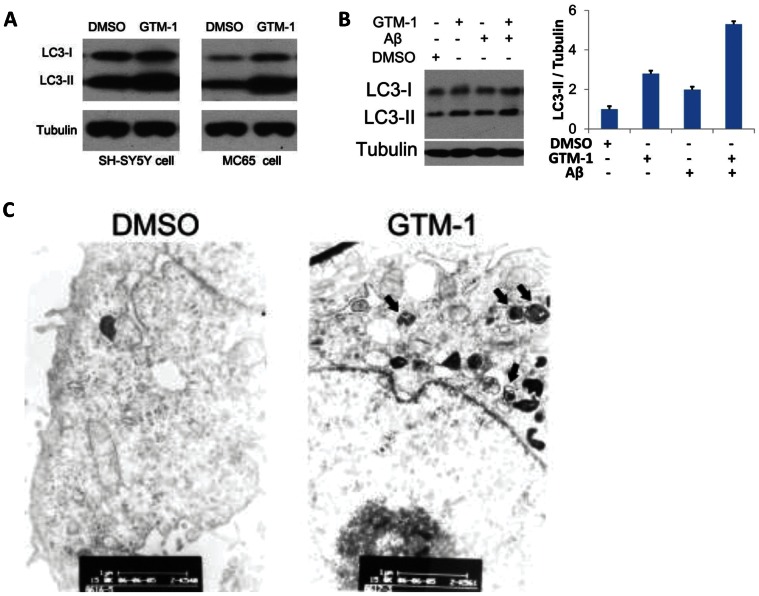
GTM-1 up-regulates autophagy level. A, SH-SY5Y/LC3-GFP and MC65 cells were treated with 20 µM GTM-1 for 12 hrs. Next, the cell lysates were analyzed using western blotting with anti-LC3 and anti-β-tubulin (as a control). B, Left: SH-SY5Y and MC65 cells were treated with 20 µM GTM-1 for 6 hrs. Next, the cell lysates were analyzed using western blotting with anti-LC3 and anti-β-tubulin (as a control). Right: quantification of the Western blot images of the ratio between LC3-II and tubulin. C, MC65 cells were treated with vehicle control (1% DMSO) or GTM-1 (20 µM) for 8 hrs. The cells were then fixed with glutaraldehyde and prepared for EM analysis. Bar, 1∶11,000. Arrows indicate double and multi-membrane autophagosomic vesicles.

To confirm the autophagy stimulation of GTM-1, SH-SY5Y and MC65 cells treated with GTM-1 were assayed by LC3 western blotting. Treatment with GTM-1 showed a robust LC3-II band (Fig. 2A), which was consistent with the HCS assay results of LC3-GFP puncta (Fig. 1B, 1C, 1D). Further studies performed in SH-SY5Y cells showed that GTM-1 can stimulate autophagy in the presence of Aβ42 (Fig. 2B).

These results were further confirmed using electron microscopy (Fig. 2C). SH-SY5Y cells treated with GTM-1 showed a large number of autophagosomes with characteristic double membranes compared to the negative control when the cells were incubated with 0.1% DMSO.

### GTM-1 Suppresses Intracellular Oxidative Stress and the Aggregation of Aβ Oligomers

MC65 is a human neuroblastoma cell line that conditionally expresses C99, a 99-residue carboxyl terminal fragment of APP, which is subsequently cleaved by c-secretase to generate Aβ [42]. Removal of tetracycline (Tet−) from MC65 culture medium resulted in the accumulation of Aβ oligomers that were subsequently processed to increase intracellular oxidative stress [30], [31].

**Figure 3:**
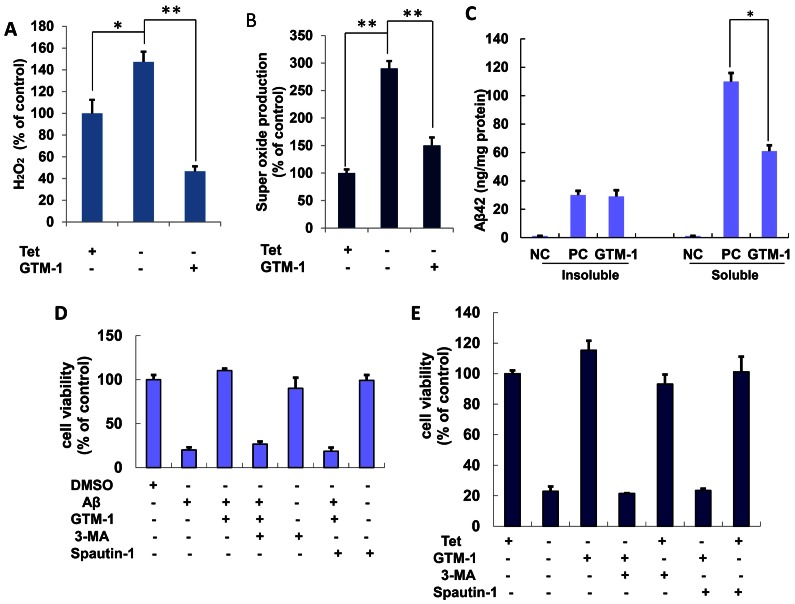
Protective effects of GTM-1 in neuroblastoma cells. A–C, MC65 cells were grown in the presence (Tet+) or absence (Tet−) of tetracycline and under Tet− with GTM-1 for 24 hrs, and assessed for: (A) H_2_O_2_ production, (B) superoxide production. C, The effect of GTM-1 on the insoluble or soluble Aβ oligomers level were assessed using ELISA in MC65 cells 72 hrs after tetracyline withdrawal (C). Cells incubated with tetracycline (Tet+) were used as a negative control (NC), and cells cultured without tetracycline (Tet−) were used as a positive control (PC). D, SH-SY5Y cells were incubated with the indicated compounds for 24 h. The cell viability was assayed using the MTT assay. Aβ, Aβ42 (30 µM), 3-MA (10 µM), spautin-1 (10 µM), GTM-1 (20 µM). Cells treated with vehicle (0.1% DMSO) were used as a negative control (NC). E, MC65 cells were grown in the presence (Tet+) or absence (Tet−) of tetracycline and under Tet− with the indicated compounds for 24 hrs, and the cell viability was assessed using the MTT assay. 3-MA (10 µM), spautin-1 (10 µM), GTM-1 (20 µM). Cells cultured in the presence of tetracycline (Tet+) were used as a negative control (NC). Stars indicated significant differences between the model group and control or treatment groups for specific time points. *P<0.05, **P<0.01.

Consistent with these reports, deprivation of tetracycline (Tet−) from the MC65 culture medium caused an increase in the intracellular H_2_O_2_ levels to 150% and an increase in super oxidation to 270% (Fig. 3A, 3B). In contrast, treatment with GTM-1 (100 µM) blocked the upregulation of super oxidation levels by deprivation of tetracycline and greatly reduced intracellular hydrogen peroxide levels (Fig. 3A, 3B). Specifically, MC65 cells grown in the presence of tetracycline (Tet+) were used as a control, and in all instances, the measurement of Tet+ MC65 cells was considered 100% and was used as a reference to which all other treatments were compared.

The effects of GTM-1 on Aβ oligomers in the soluble and insoluble cell fractions were assessed using ELISA. As shown in Figure 3C, in the negative control (NC), no Aβ oligomers could be detected. After tetracycline withdrawal (Tet−), the Aβ oligomers accumulated to 310 pg/ml in the insoluble fraction and 1100 pg/ml in the soluble fraction. GTM-1 treatment clearly reduced the accumulation of Aβ oligomers caused by tetracycline withdrawal in the soluble cell fraction. In the insoluble cell fraction, the Aβ oligomer levels were similar between the samples treated with or without GTM-1 (Fig. 3C).

### Autophagy is Necessary for GTM-1 Neuroprotection from Aβ Toxicity

To better understand the relationship between GTM-1, autophagy and Aβ42 toxicity, we performed a combination treatment test. Specifically, SH-SY5Y cells were treated with GTM-1 and Aβ42. Treatment with extracellular Aβ42 showed toxicity and affected cell growth, whereas treatment with GTM-1 antagonized the effects of Aβ oligomers. In contrast, in the presence of the autophagy inhibitor 3-methyladenine (3-MA) and spautin-1, GTM-1 failed to protect neurons from Aβ oligomers, while treatment with 3-MA or spautin-1 had little effect on SH-SY5Y cell growth (Fig. 3D).

A similar combination treatment test was performed in MC65 cells. MC65 cells incubated without tetracycline (Tet−) were used as a positive control, which expressed an accumulation and precipitation of Aβ, resulting in cell death. Furthermore, GTM-1(20 µM) clearly reduced Aβ-induced toxicity. In contrast, GTM-1 failed to protect neurons against Aβ toxicity in the presence of the autophagy inhibitor, 3-MA or spautin-1 (Fig. 3E).

### GTM-1 Modulates Autophagy in Neurons using a Different Mechanism Compared to Rapamycin

**Figure 4:**
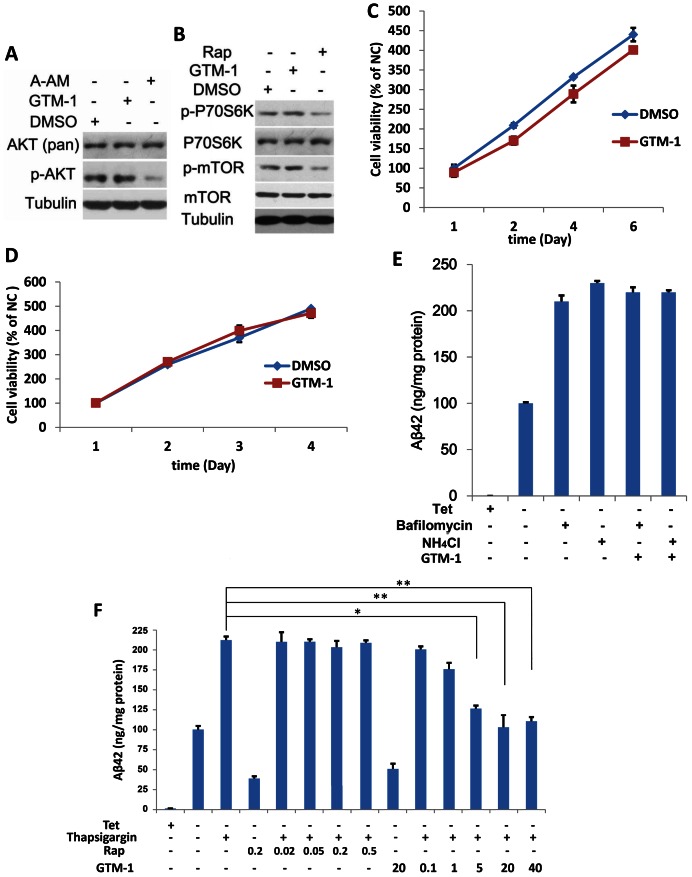
GTM-1 modulates autophagy in neurons using a different mechanism from rapamycin. A–B, SH-SY5Y cells were treated with the indicated compounds for 12 hrs. Next, the cell lysates were analyzed using western blotting with specific antibodies. DMSO (0.1%), GTM-1 (20 µM), Rap: Rapamycin (0.2 µM), A-AM (0.1 µM). β-tubulin was used a control. C–D, SH-SY5Y (4C) or MC-65 (4D) cells were incubated with GTM-1 (20 µM) for the indicated times. Cells treated with vehicle (0.1% DMSO) for 24 hrs were used as a negative control (NC). Cell viability was assessed using MTT, and all of the data were normalized to the NC. E, MC65 cells were grown in the presence (Tet+) or absence (Tet−) of tetracycline and under Tet− with GTM-1 (20 µM) for 8 hrs. The indicated compounds were then added within 2 hrs. Soluble Aβ oligomers were assessed using ELISA in MC65 cells. Bafilomycin (100 nM), NH_4_Cl (10 mM). F, MC65 cells were grown in the presence (Tet+) or absence (Tet−) of tetracycline and under Tet− with GTM-1 (at indicated concentrations) for 5 hrs, and the indicated compounds were added within 5 hrs. The soluble Aβ oligomers were assessed using ELISA. Rap: Rapamycin (at the indicated concentrations), thapsigargin (3 µM). Stars indicate significant differences between the model group and control or treatment groups for specific time points. *P<0.05, **P<0.01.

Previous reports have shown that mTOR is hyperactive in select neurons in AD brains, although the link between mTOR hyperactivity and AD progression is still unclear. Rapamycin and several other autophagy inducers, such as curcumin [32], have been found to induce autophagy by inhibiting the Akt/mTOR/p70S6K pathway. To test whether GTM-1 exhibits a similar ability to inhibit Akt, the phosphorylation levels of Akt (phosphoryl-threonine 308) were assayed using the known Akt inhibitor A-AM (Sigma) as a positive control [33]. As shown in Figure 4A, in contrast to A-AM, GTM-1 treatment had no effects on the phosphorylation levels of Akt. Further assays also demonstrated that GTM-1 induced autophagy, but did not stimulate phosphorylation of mTOR or p70S6K (Fig. 4B). These results demonstrated that GTM-1 upregulated autophagy in neurons in an Akt- and mTOR independent manner.

Autophagy may also be promoted by several toxic compounds [34]. To determine if GTM-1 demonstrates some influence on neuronal growth, resulting from its effects on intracellular autophagy, we measured the viability of SH-SY5Y cells treated with GTM-1 (Fig. 4C) using MTT assays. Our results showed that the cell viability after GTM-1 treatment was similar to the negative-control cells (Fig. 4C). These results were consistent with our findings obtained using MC65 cells (Fig. 4D). These results showed that GTM-1 upregulates autophagy in neurons in the absence of cytotoxicity.

Lysosomal disorder or autophagosome-lysosome fusion blocking also disturbs autophagy flux [17], [18]. To test whether GTM-1 has an effect on these processes, two lysosome inhibitors (NH_4_Cl and Bafilomycin A1) and two small molecules that disrupt autophagy-lysosome fusion (thapsigargin and asparagine) were used. Consistent with previous reports, NH_4_Cl and Bafilomycin A1 inhibited lysosome function and subsequently inhibited autophagy clearance efficiency of Aβ, while neither GTM-1 nor rapamycin could reverse the inhibition of NH_4_Cl or Bafilomycin A1 on autophagy flux (Fig. 4E). Furthermore, thapsigargin and asparagine, two lysosome-autophagosome inhibitors, caused clear accumulation of Aβ in MC56 cells with the indicated treatment conditions (Fig. 4F and Fig. S2). Importantly, GTM-1 could reverse the inhibition of autophagy efficiency by thapsigargin and asparagine, which differed from that of rapamycin (Fig. 4F and Fig. S2).

To determine the effect of GTM-1 on lysosomal stability, LysoTracker Red, a lysosomotropic fluorescence probe, was used. GTM-1 had no obvious effect on lysosome number and distribution (Fig. S3).

Taken together, GTM-1 may target the maturation and fusion steps of autophagy.

### Tolerance Paradigm Assay

**Figure 5:**
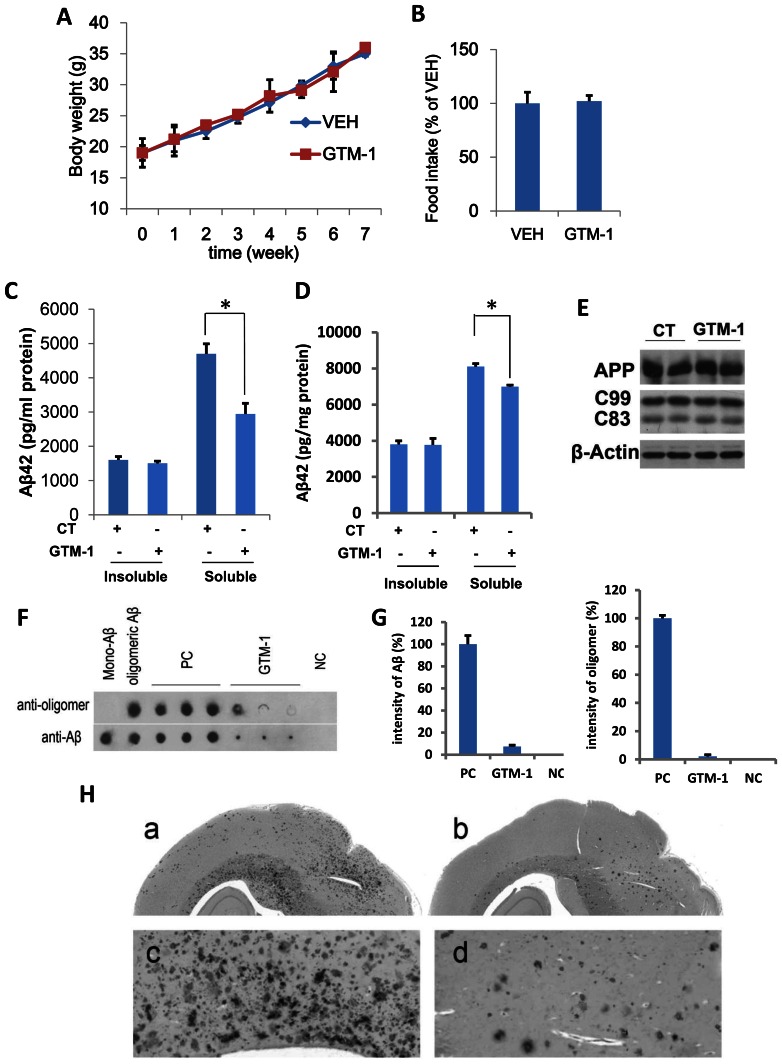
GTM-1 rescues Aβ and Tau pathology in AD mice. A–B, GTM-1 (6 mg/kg/day) or vehicle (VEH) was administered to five-month-old 3XTg-AD mice daily for 8 weeks. The average body weight (A) and total food consumed for 8 weeks (B) was recorded. C–D, F–G, 5-month-old 3XTg-AD mice (C, D) or 15-month-old 3XTg-AD mice (F, G) were treated with GTM-1 (3 mg/kg/day) daily for 8 weeks. The insoluble and soluble Aβ42 (C, D) levels were measured using a sandwich ELISA. E, Brain proteins were obtained from 8 different 3xTg-AD mice treated with GTM-1 and 8 different 3xTg-AD mice treated with vehicle after 8 weeks of treatment. The endogenous expression of APP, C99 and C83 were assessed using western blotting analyses. CT (control treatment) mice fed with a normal (vehicle) diet. Stars indicate significant differences between the model group and control or treatment groups for that time point. *P<0.05, **P<0.01. F, Five-month-old 3XTg-AD mice were treated with GTM-1 (6 mg/kg/day) or vehicle (PC, positive control) for 2 months. The brain sample from non-Tg mice with the same generic background was used as a negative control (NC). Soluble fractions extracted from brain samples were applied onto Hybond C-extra membrane. Next, the bound oligomeric Aβ and mono-Aβ on the membranes were detected using mono-Aβ specific antibodies, and oligomer-specific antibodies, respectively. Commercial soluble Aβ monomers and oligomeric Aβs produced from the monomer were spotted as indicated and used as another control. G, Densitometric analysis of the immune dot-blot experiments was performed using a high-resolution scanner, and the average intensity of each group was normalized using the average PC result. H, Five-month-old 3XTg-AD mice were treated as previously. Representative sections obtained from the brains of mice treated with GTM-1 and vehicle were immunostained using an Aβ-specific antibody.

Cellular experiments in SH-SY5Y and MC65 cells demonstrated that GTM-1 had no toxic effect on neurons (Fig. 3C, 3D). To further evaluate the toxicity of GTM-1, the effects of GTM-1 on animal body weight were measured in WT mice. The body weights of the mice were measured at different time points during the 8-week treatment period, and the results are shown as weekly averages for groups of vehicle (VEH)- and GTM-1 (6 mg/Kg/day)-treated mice. As shown in Figure 5A and 5B, the body weights of VEH- and GTM-1 (6 mg/Kg/day)-treated animals remained similar during most of the treatment period. Moreover, no mice died during the assay period. In addition, the total food obtained by the vehicle (VEH) and GTM-1-treated groups were not obviously different in two months (Fig. 5B).

### GTM-1 Rescues Aβ Pathology in AD Mice

Accumulation of Aβ is an invariant feature of AD. To better understand the effects of GTM-1 on protein accumulation in vivo, we measured the Aβ levels in different brains of 5-month-old 3×Tg-AD mice. After 2 months of daily administration of GTM-1 (3 mg/kg/day), the mice were sacrificed, and their brains were isolated for biochemical assays. As controls, 3×Tg-AD mice were fed a normal diet. We first analyzed the effect of GTM-1 treatment on Aβ deposition. Soluble Aβ42 levels were reduced by 37% in the group treated with GTM-1 compared to the group treated with vehicle (Fig.5C). Moreover, GTM-1 selectively decreased soluble Aβ, but had no obvious effect on insoluble Aβ (Fig. 5C).

To further explore the regulatory mechanism of the reduction in Aβ by GTM-1 in vivo, we subsequently examined if the decrease in Aβ levels was due to changes in its production. The endogenous expression levels of APP, C99 and C83 in brain samples were assessed using western blotting analyses. The levels of APP, C99 and C83 were similar between the mice with or without GTM-1 administration, indicating that GTM-1 did not affect APP processing and therefore Aβ production (Fig. 5E).

In addition, it was reported that rapamycin failed to enhance the degradation of Aβ oligomers in 15-month old AD mice [5]. To assess whether GTM-1 could reverse the Aβ pathology in older mice, 15-month-old 3×Tg-AD mice were treated with GTM-1 (3 mg/Kg/day) daily for 2 months. As a negative control, 15-month-old 3×Tg-AD mice treated with vehicle were used. GTM-1 caused a 21% reduction in the levels of soluble Aβ42 in the brain of 15-month-old 3×Tg-AD mice (Fig. 5D). In addition, 8% of the older mice died in the control group, while no mice died in the GTM-1 treatment group.

Furthermore, we analyzed the effect of chronic GTM-1 administration on mTOR signaling. GTM-1 induced autophagy but did not stimulate activation by phosphorylation of mTOR or p70S6K in vivo (Fig. S4), which was consistent with the results obtained from the MC65 cell assay.

To further explore the effect of GTM-1 on Aβ deposition, dot blot assays were used to identify the assembled forms of Aβ in fresh mice brain samples. AD samples were compared with age-matched control tissue. The antibody reactivity was robust in AD brain extracts for both the oligomer conformation- and Aβ sequence-specific antibodies compared to the WT sample, indicating an alteration in Aβ oligomerization in the model mouse brains (Fig. 5F). In addition, immunoreactivity was reduced in the group treated with GTM-1, which indicated that the oligomeric Aβ in the AD mice was clearly reduced after 2 months of GTM-1 treatment. Densitometric analysis of the immune dot-blot experiments showed consistent results (Fig. 5G).

We also determined whether the Aβ plaque load was altered by GTM-1 treatment using immunohistochemistry. Sections obtained from 3×Tg-AD mice treated with GTM-1 or vehicle were stained with an anti-Ab42 specific antibody [5]. The 3×Tg-AD mice showed widespread Aβ deposition throughout the brain (Fig. 5H-a, c). In contrast, Aβ deposition was reduced in the 3×Tg-AD mice treated with GTM-1 (Fig. 5H-b, d). Taken together, these results demonstrate a reduction of Aβ deposition induced by GMT-1 treatment.

### GTM Administration Enhances Learning and Memory Ability in AD Mice

To determine whether the reduction of aggregated Aβ by GTM-1 contributes to the cognitive phenotype observed in 3×Tg-AD mice, we used the Morris water maze. Five-month-old 3×Tg-AD mice were given normal food or food containing GTM-1 daily for two months, and non-Tg mice were used as controls. The escape latency of each animal group was correspondingly shortened as training progressed, suggesting that the mice had gradually mastered how to find the platform during training (Table 1). Compared to the normal group, the escape latency of the model group was significantly extended (P<0.01) (Table 1); in contrast, the escape latency of the GTM-1 treatment group was significantly shorter compared to the model group (P<0.05). Taken together, these results indicate that GTM-1 administration rescued the learning and memory deficits in the AD mice and thus highlights the potential efficacy of GTM-1.

**Table 1:** The escape latency of the mice in the Morris water maze test (x ± s) (n = 12).

Group	The escape latency				
	Day1	Day2	Day3	Day4	Day5
NS control group	33.67±19.96**	37.08±17.03**	37.58±14.76**	37.58±15.78**	33.58±15.05**
AD model group	67.92±25.13	71.17±22.20	70.67±21.15	68.83±25.17	67.75±23.80
Treatment group	50.75±13.38*	50.58±18.58*	46.75±17.62**	44.17±14.93**	40.42±17.40**

Five-month-old 3xTg-AD and non-Tg mice were on a mixed C57Bl6/129 background. The non-Tg mice were used as the NS control group and the 3xTg-AD mice were used as the AD model group. The 3xTg-AD mice treated with GTM-1 (3 mg/kg/day) daily for two months were included in the treatment group in this assay. The escape latency of the mice was recorded every day. Stars indicate significant differences between the model group and control or treatment groups for specific time points.

* P<0.05,

** P<0.01.

## Discussion

In this study, we identified a novel autophagy inducer, GTM-1, using a two-cycle screening strategy based on imaging high-content screening (HCS) and cell toxicity assay, which rapidly and efficiently induced autophagy in neurons. Our study demonstrated that GTM-1 modulated autophagy in an Akt-independent and mTOR-independent manner, and using a different mechanism from rapamycin. Importantly, GTM-1-mediated upregulation of autophagy attenuated Aβ pathology and ameliorated cognitive deficits in Alzheimer’s disease (AD) mice.

As previously described, there have been several lines of research focused on exploiting small molecules targeting autophagy [19]–[22], such as rapamycin and SMER28. In particular, of these molecules, rapamycin has been the most extensively studied compound in terms of its role in regulating autophagy in neurodegenerative disorders, and accumulated evidence has shown that prophylactically rapamycin can abolish AD pathology in many cases [5], [20], [26]. However, other evidence has shown that once Aβ plaques are well established throughout the brain of old mice (15 months old), increasing autophagy induction by rapamycin is not sufficient to rescue AD-like pathology and its associated deficits [5].

It is well documented that age-dependent decreases in autophagy may contribute to the accumulation of protein in the brain and in neurodegenerative disorders. However, the effect of rapamycin in increasing autophagy induction in old AD mice cannot degrade Aβ oligomers as suggested by Smita. M. et al. [5]. However, these studies will provide us with a better understand of AD pathology. To further explore this question, we identified a novel autophagy inducer, which uses a different autophagy-regulation mechanism from rapamycin (Fig. 3J).

It is well known that the autophagy process can be divided into three key steps: autophagy induction, vesicle nucleation and expansion, and autophagosome formation [17], [18]. Rapamycin increases autophagy induction via inhibition of mTOR, which negatively regulates autophagy induction through super-phosphorylation of Atg13 and inhibition of autophagosome nucleation [17]. Thus, rapamycin mainly affects the induction of autophagy, which is “upstream” of the autophagic process. This “upstream” elevation can be completely inhibited by thapsigargin and asparagine, inhibitors of autophagosome-lysosome fusion, which is the “downstream” step of the autophagic process [35], [36].

A growing number of studies have found that disruption of autophagy-lysosome fusion rather than impairment of autophagy induction in the neurons results in protein accumulation in AD brains and in AD mouse models [45]. Moreover, accumulated Aβ oligomers have been implicated in the modulation of autophagy, despite being an autophagy substrate [26]. Moreover, exogenous expression of intracellular Aβ results in the inhibition of autophagosome maturation [37], [38]. In addition, excessive amounts of intracellular Aβ can lead to a disruption of the lysosomal degradative system [39], [40].

The functional role of Aβ oligomers on autophagy is relatively similar to the role of thapsigargin and asparagine, in that they all inhibit “downstream” of the autophagy flux. Thus, an alternate hypothesis may be put forward that once plaques are well established in the brains of old AD mice and subsequently inhibit autophagy flux, stimulation of autophagy by rapamycin cannot suffice to reverse this accumulation in protein but only contributes to increasing autophagosome accumulation [6]. Thus, when evaluating a potential autophagy targeting therapeutic agent in cellular or mice AD models, it is critical to assess its effects not only on the induction of autophagy but also on the enhancement of autophagy flux and autophagy clearance efficiency.

Thapsigargin and asparagine are two specific inhibitors of lysosome-autophagy fusion. Lan et al. found that thapsigargin specifically blocked the fusion of autophagosomes and lysosomes while leaving the endocytic system functional [35]. Another inhibitor, asparagine, did not affect autophagosome formation but did lead to the accumulation of mature autophagosomes via blockade of autophagosome fusion and the endocytic system [36]. In this study, we employed these two inhibitors to interrupt the “downstream” autophagy flux. Unexpectedly, in contrast to rapamycin, GTM-1 reversed the inhibition of autophagy efficiency by thapsigargin and asparagine (Fig. 4F and Fig. S2), conferring neuroprotection. Moreover, it showed a promising reduction in Aβ and it enhanced learning and memory in 15-month-old AD mice treated with GTM-1 (Fig. 5D and Table 1). The different action of GTM-1 on 15-month-old mice showed that the established Aβ plaques did not clearly suppress the enhancement of autophagy clearance induced by GTM-1.

In addition, our study showed that inhibiting lysosome function using the small molecules, NH_4_Cl and Bafilomycin A1, could block the ameliorating effects of GTM-1 on Aβ accumulation (Fig. 4E). Degradation of the engulfed material was primarily mediated by lysosomal enzymes that optimally functioned within a narrow range of acidic pH values. High concentrations of NH_4_Cl (10 mM), an alkaline compound, could robustly elevate lysosomal pH (pHL) and inhibit proteolysis in the lysosome. In this study, we demonstrated that when lysosome degradation was abolished, GTM-1 could not enhance autophagy clearance (Fig. 4E). Immunofluorescence staining studies also showed that GTM-1 had no clear effect on the lysosome (Fig. S3).

In addition, 3-MA, which inhibits autophagy induction by blocking Vps34 kinase activity, deterred the effects of GTM-1 on SH-SY5Y and MC65 cells (Fig. 3D, 3E). Similar to 3-MA, Spuatin-1, which inhibits autophagy induction by increasing Beclin1 degradation, also blocked the effect of GTM-1.

Taken together, these observations support the proposal that GTM-1 may target the maturation and fusion steps of autophagy, which may be critical in AD development.

Using GTM-1 as a tool, we explored its potential restorative effects on autophagy efficiency in 3xTg-AD mice, a well-established Alzheimer’s disease rodent model [6].

After 2 months of oral administration, promising therapeutic effects on Aβ pathology and cognitive deficits were obtained, demonstrating the potential of downstream autophagy flux modulation as a therapeutic strategy. Further investigation of whether the drug-sensitivity of GTM-1 varies among the mice of different ages was performed. We found that 15-month-old 3xTg-AD mice that were treated with GTM-1 were similar to 5-month-old mice, and the accumulation of dysfunctional protein in the brain was also clearly reduced.

Furthermore, GTM-1 administration reduced Aβ levels in neurons in AD mice, while the levels of APP, C99 and C83 were similar between the two groups with or without GTM-1 feeding, indicating that GTM-1 had little effect on APP processing and subsequent Aβ production. However, other targeting regulation factors or processes controlled by GTM-1 may exist, such as direct enhancement of autophagy degradation of Aβ oligomers.

Although great caution must be applied when extrapolating from findings obtained from experimental models to complex human diseases, our results demonstrated that induction of autophagy by GTM-1 might present a potential therapeutic strategy in AD. Moreover, GTM-1 might be a promising lead compound for the development of novel anti-AD drugs, although the exact mechanism by which GTM-1 modulates autophagy in neurons still requires further investigation.

## Materials and Methods

### Reagent and Antibody

Rapamycin, A-AM, NH_4_Cl, thapsigargin, asparagine and bafilomycin A1 were purchased from Sigma (Sigma, St. Louis, Missouri). Aβ42 was purchased from AnaSpec (AnaSpec, Inc., San Jose, California). To make the mono-Aβ solution, Aβ42 powder was re-suspended in PBS buffer (containing 0.2% NH_4_OH and 0.1% DMSO) at a concentration of 30 µM with shaking at 25°C. Fibrils were pelleted by spinning the assembly mixtures at 113,000 × g for 20 min. The supernatant was sonicated for 2 minutes prior to use. To generate Aβ oligomers, Aβ42 powder was dissolved in 1% NH_4_OH at 2 mg/mL, and the pH value was adjusted to 7.0 using 1 M HCl. The mixture was kept at 37°C without shaking for 7 days to induce peptide aggregation as previously described [43]. Rabbit polyclonal antibody anti-LC3 and mouse monoclonal antibody anti-β-tubulin were obtained from Sigma. Rabbit polyclonal anti-oligomer antibody A11 (cat. AHB0052) and mouse monoclonal antibody anti-mono Aβ (cat. AMB0062) were purchased from Invitrogen (Camarillo, CA). Aβ42 levels from the soluble and insoluble fractions were measured using an ELISA kit purchased from Invitrogen. The SH-SY5Y and MC65 cell lines were obtained from the Cell Bank of the Chinese Academy of Sciences (Shanghai, China). The H-NMR spectrum was assessed at the Department of Chemistry in Fudan University (Fig. S1).

### Cell Cultures and the Selection of GFP-LC3 Stable Clone

MC65 cells were maintained using 5% CO_2_ at 37°C, which was incubated with 10% fetal bovine serum (FBS; Biological Industries, Grand Island, New York), 2 mM L-glutamine and 1 µg/mL tetracycline (Tet) as previously described [4], [31]. Cell toxicity was produced by inducing C99 expression after removing Tet. We limited the assays to early cell passages. The cell viability was measured using a colorimetric MTT assay because the results were comparable with the data obtained by quantifying the viable cells on the basis of trypan blue exclusion and the live/dead assay [30], [31].

SH-SY5Y cells were cultured with 5% CO_2_ at 37°C in F12/MEM growth medium supplemented with 10% fetal bovine serum, 4,500 mg/L glucose, 100 U/mL penicillin and 0.1 mg/mL streptomycin (Invitrogen, Carlsbad, California). SH-SY5Y cells were transfected with pEGFP-LC3 or pEGFP (pEGFP-C1; Clontech, Palo Alto, California) with 0.1% lipofectamine 2000 reagent (Invitrogen, Carlsbad, California). A stable clone of SH-SY5Y/pEGFPLC3 cells was selected for the following studies as previously described [29].

### Protein Extraction and Elisa Assay

After the mice were sacrificed using CO_2_ asphyxiation, the brains were extracted and frozen in dry ice. At 4°C, the frozen brains were homogenized in a tissue extraction reagent containing 0.7 mg/ml Pepstatin A supplemented with complete Mini protease inhibitor tablets (Roche, Gipf-Oberfrick, Switzerland) and phosphatase inhibitors (1∶100, Calbiochem). The lysates were sonicated to sheer the DNA, and centrifuged at 4°C for 1 h at 100,000 × g. The supernatant was then stored as a soluble fraction. The pellet was re-homogenized in 70% formic acid and centrifuged as previously described. The supernatant was then stored as the insoluble fraction [6]. The protein concentrations were determined using the bicinchoninic acid (BAC, Pierce) assay.

The MC65 cells were homogenized in RIPA containing 0.7 mg/ml Pepstatin A supplemented with complete Mini protease inhibitor tablets and phosphatase inhibitors. The lysates were sonicated and centrifuged at 4°C for 1 h at 100,000 × g, and the supernatants were maintained as a soluble fraction. The pellets were washed with RIPA and resuspended in 70% formic acid and centrifuged as previously described. The supernatants were then saved as an insoluble fraction. The protein concentrations were determined using the bicinchoninic acid assay.

Aβ42 levels from the soluble (see above) and insoluble fractions were measured using a sensitive sandwich ELISA system according to previously described protocols [38].

### Quantification of EGFP-LC3-II-positive Punctate Dots

The SH-SY5Y/pEGFP-LC3 cells were seeded in 96-well plates (Constar), treated with Aβ42 (10 µM) peptides with or without GTM-1 (20 µM) at specific time points, and then fixed with 4% paraformaldehyde (Sigma) and stained with 3 mg/ml DAPI (Sigma). Images were collected using an ArrayScan HCS 4.0 Reader with a 20× objective (Cellomics ArrayScan VTI) for DAPI-labeled nuclei and GFP-tagged intracellular proteins. The Spot Detector BioApplication was used to acquire and analyze the images after optimization. Images were obtained from 1,000 cells for each compound treatment and analyzed to obtain the average GFP spot intensity per cell [25]. DMSO and rapamycin were used as the negative and positive control, respectively.

### Determination of Free Radicals

Two days after the cells were seeded, the level of intracellular superoxide radicals was determined in the cell lysates using the nitroblue tetrazolium (NBT) assay as previously described [30]. NBT (Sigma, St. Louis, MO) was added to the cell media at a final concentration of 1 mg/ml and incubated for 3 h at 37°C in a humidified atmosphere containing 5% CO_2_. The NBT-containing medium was removed, the cells were washed twice with warm PBS followed by methanol, and then air-dried. Intracellular blue formazan particles were dissolved by adding 120 µl/well 2 M potassium hydroxide followed by 140 µl/well DMSO. The absorbance of the dissolved NBT was measured at 620 nm using a Spectra Max Plus (Thermo Fisher).

The hydrogen peroxide (H_2_O_2_) and intracellular superoxide levels were measured using previously described methods [30]. Briefly, the H_2_O_2_ levels were determined in the cell media collected from the cells treated with Tet+, Tet−, and Tet− PA for 48 hrs using the Bioxytech H_2_O_2_-560TM Quantitative Hydrogen Peroxide Assay (Oxis International, Inc., Foster City, CA), which was performed according to the manufacturer’s instructions.

### Mice and Compound Treatment

Derivation and characterization of the 3×Tg-AD mice has been described elsewhere [6]. The 3×Tg-AD mice were maintained by hybridization with C57Bl6/129 mice (Tianjin, Biotech.). Briefly, transgenes encoding human APP_Swe_ were microinjected into single-cell embryos harvested from homozygous mutant PS1_M146V_ knock-in (PS1-KI) mice. The 3×Tg-AD and non-Tg mice used in these studies were bred on a mixed C57Bl6/129 background. The mice were maintained in a climate-controlled environment with a 12-hr light/12-hr dark cycle. All of the procedures were performed in accordance with the Guidelines for the Care and Use of Laboratory Animals of Fudan University.

The assay compounds were administered to the mice in a facilitated delivery method as previously described [41]. Briefly, GTM-1 was microencapsulated and used at a dose of 14 mg per kg of food (verified by HPLC). On the assumption that the average mouse weighs 30 mg and consumes 5 mg of food/day, the mouse was given 2.3 mg GTM-1 per kg body weight/day. All of the mice were given ad libitum access to GTM-1 or control food and water for the duration of the experiment.

### Morris Water Maze Test

The Morris water maze test was performed after 3 weeks of drug administration. GTM-1 (6 mg/kg) was administered to the 3×Tg-AD mice every day for two months. The Morris water maze testing apparatus consisted of a circular water pool and an automatic picture recording and analysis system. A camera was placed over the pool, which was connected to a computer. When the preset training time (120 s) was over or the animal had reached the platform, the computer would stop tracking the animal and recorded the swimming track. In these experiments, clean water was placed into the pool, and milk powder was added to make the water white and opaque. The platform was placed approximately 1.5 cm under the water, and the water temperature was maintained between 18°C and 22°C. The mice were placed into the water from different quadrants once a day. If the mouse did not find the platform within 120 s, then the mouse was manually placed onto the platform and kept there for 10 s. If the mouse did find the platform within 120 s, then it was also kept on the platform for 10 s. After training, the mice were wiped dry and put back into their cages. Three days after the first training, the time spent finding the platform (referred to as the escape latency) by the mice was recorded in the following 5 days.

### Immunoassay for Oligomeric Aβ

Oligomeric Aβ blot assays were performed as previously reported [44]. Briefly, fresh cortical tissue was homogenized in the absence of detergent in lysate buffer containing 0.7 mg/ml Pepstatin A supplemented with complete Mini protease inhibitor tablets (Roche, Gipf-Oberfrick, Switzerland) and phosphatase inhibitors (1∶100 Calbiochem). Soluble fractions contained clear supernatants that were centrifuged at 100,000×g 20-min spins at 4°C. The supernatants were spotted onto Hybond C-extra membranes (Amersham) and allowed to dry overnight in the dark. After washing away the unbound material with TBST, the non-specific sites were blocked by soaking the membranes in 5% BSA in TBST (0.5–1 hr, RT). Next, the bound oligomeric Aβs and mono-Aβs on the membrane were labeled using the mono-Aβ specific antibody (Invitrogen) and oligomer-specific antibody (Invitrogen), respectively, and were visualized using enhanced chemiluminescence.

### Immunohistochemistry

For immunohistochemical analysis, 50-mm thick sections were obtained using a Leica vibratome slicing system and the sections were stored at 4°C in 0.02% sodium azide in PBS. To quench the endogenous peroxidase activity, free-floating sections were incubated for 30 minutes in H_2_O_2_. For Aβ staining, the sections were subsequently incubated in 90% formic acid for 7 minutes to expose the epitope. The mouse monoclonal antibody anti-mono Aβ (Invitrogen, Cat. AMB0062) was applied and the sections were incubated overnight at 4°C. After removing the excess primary antibody, the sections were incubated in the appropriate secondary antibody for 1 hr at 20°C. After a 20-minute final wash, the sections were developed with diaminobenzidine (DAB) substrate using the avidin-biotin horseradish peroxidase system (Vector Labs, Burlingame, CA). Images were obtained using a digital Zeiss camera and analyzed using ImageJ software.

### Statistical Analysis

All of the values are expressed as the mean ± standard deviation (SD). The statistical significance was evaluated using Student’s t-test for paired data, and P<0.05 was considered significant.

## Supporting Information

Figure S1 **The H-NMR spectrum was assessed at the Department of Chemistry in Fudan University.**
(TIF)

Figure S2 **MC65 cells were grown in the presence (Tet+) or absence (Tet−) of tetracycline and under Tet− with GTM-1 (20 µM) for 8 hrs, and the indicated compounds were added within 2 hrs.** The soluble Aβ oligomers were assessed using ELISA in MC65 cells. Asparagine (10 mM), Rap: rapamycin (0.2 µM).
(TIF)

Figure S3 **SH-SY5Y cells treated with DMSO (0.1%) or GTM-1 (20 µM) for the indicated time points were fixed with 3.8% PFA and stained with lysotracker and observed under a fluorescence microscope.** Bar, 1∶100.
(TIF)

Figure S4 **Five-month-old 3XTg-AD mice were administered daily with GTM-1 (3 mg/kg/day) or with vehicle (WEH) for 8 weeks.** Brain proteins were obtained from 8 different 3xTg-AD mice treated with GTM-1 and 8 different 3xTg-AD mice treated with vehicle after 8 weeks of treatment, and the protein levels or protein phosphorylation levels were assessed with the indicated antibodies.
(TIF)
