# Supplementary figures and images for: Induction of Autophagy by a Novel Small Molecule Improves Aβ Pathology and Ameliorates Cognitive Deficits
Source: PLoS One. 2013 Jun 4;8(6):e65367. doi: 10.1371/journal.pone.0065367 (PMC3672196; doi:10.1371/journal.pone.0065367)

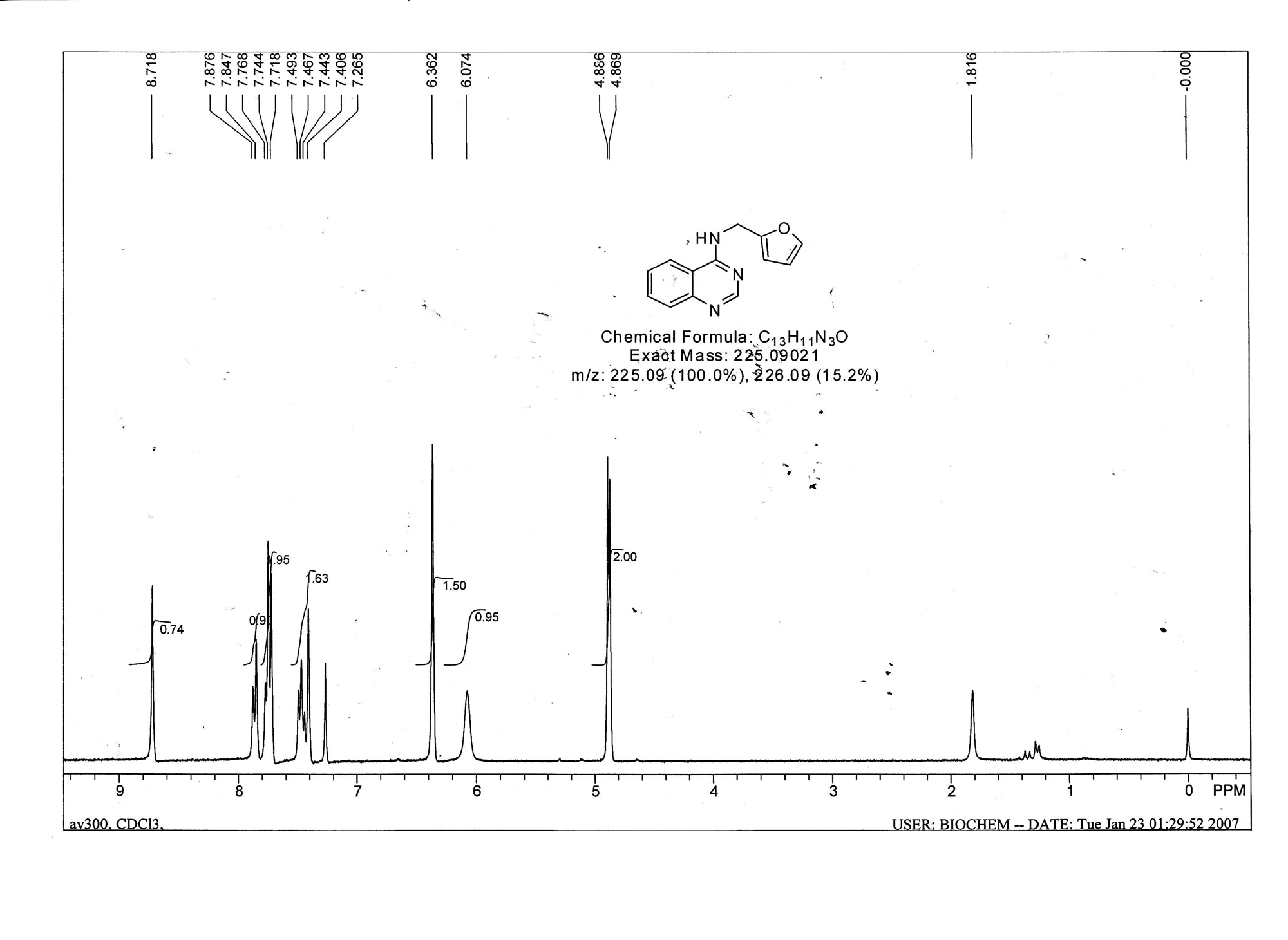

Supplement: Figure S1 — The H-NMR spectrum was assessed at the Department of Chemistry in Fudan University. (TIF) [file pone.0065367.s001.tif]

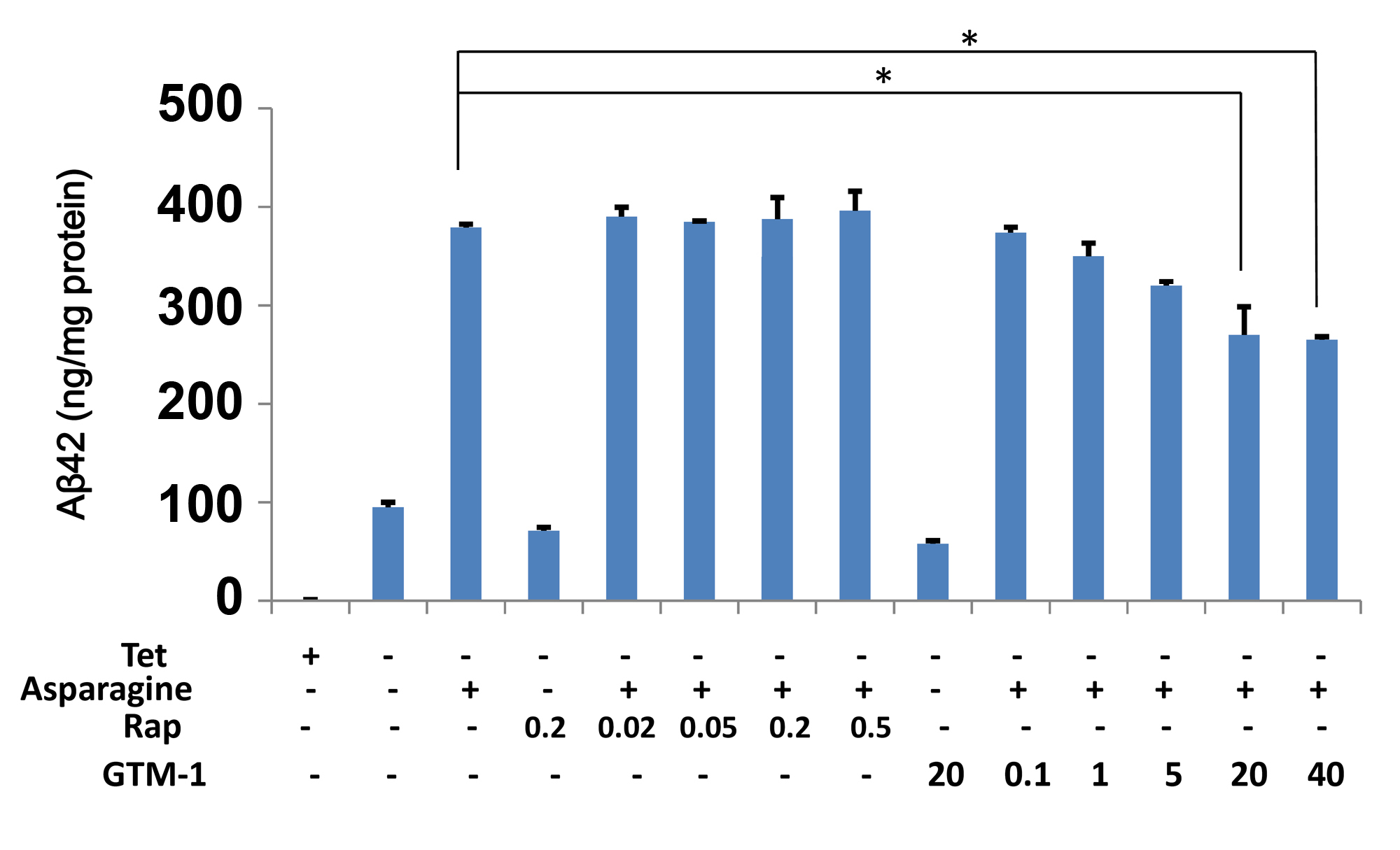

Supplement: Figure S2 — MC65 cells were grown in the presence (Tet+) or absence (Tet−) of tetracycline and under Tet− with GTM-1 (20 µM) for 8 hrs, and the indicated compounds were added within 2 hrs. The soluble Aβ oligomers were assessed using ELISA in MC65 cells. Asparagine (10 mM), Rap: rapamycin (0.2 µM). (TIF) [file pone.0065367.s002.tif]

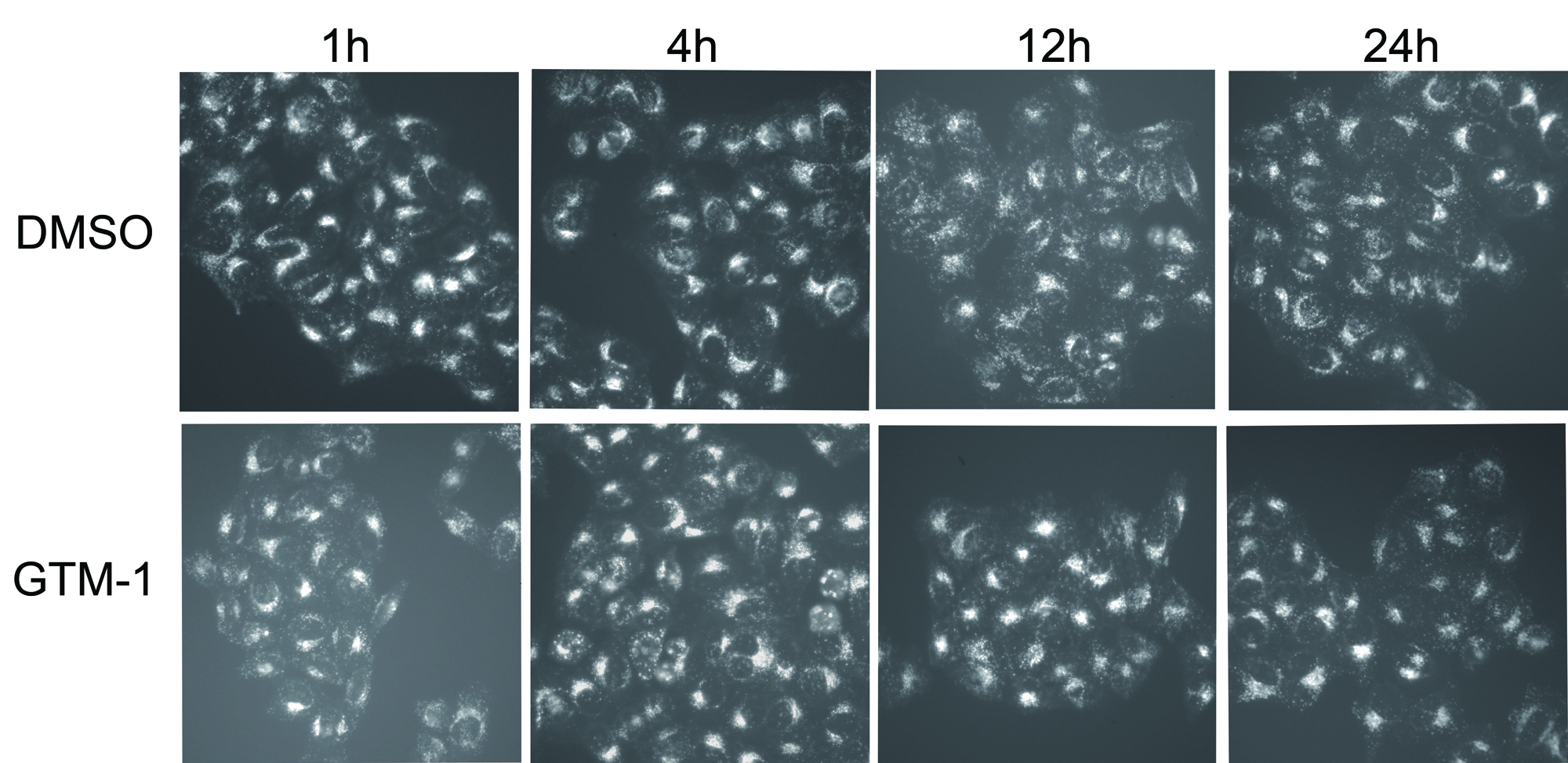

Supplement: Figure S3 — SH-SY5Y cells treated with DMSO (0.1%) or GTM-1 (20 µM) for the indicated time points were fixed with 3.8% PFA and stained with lysotracker and observed under a fluorescence microscope. Bar, 1∶100. (TIF) [file pone.0065367.s003.tif]

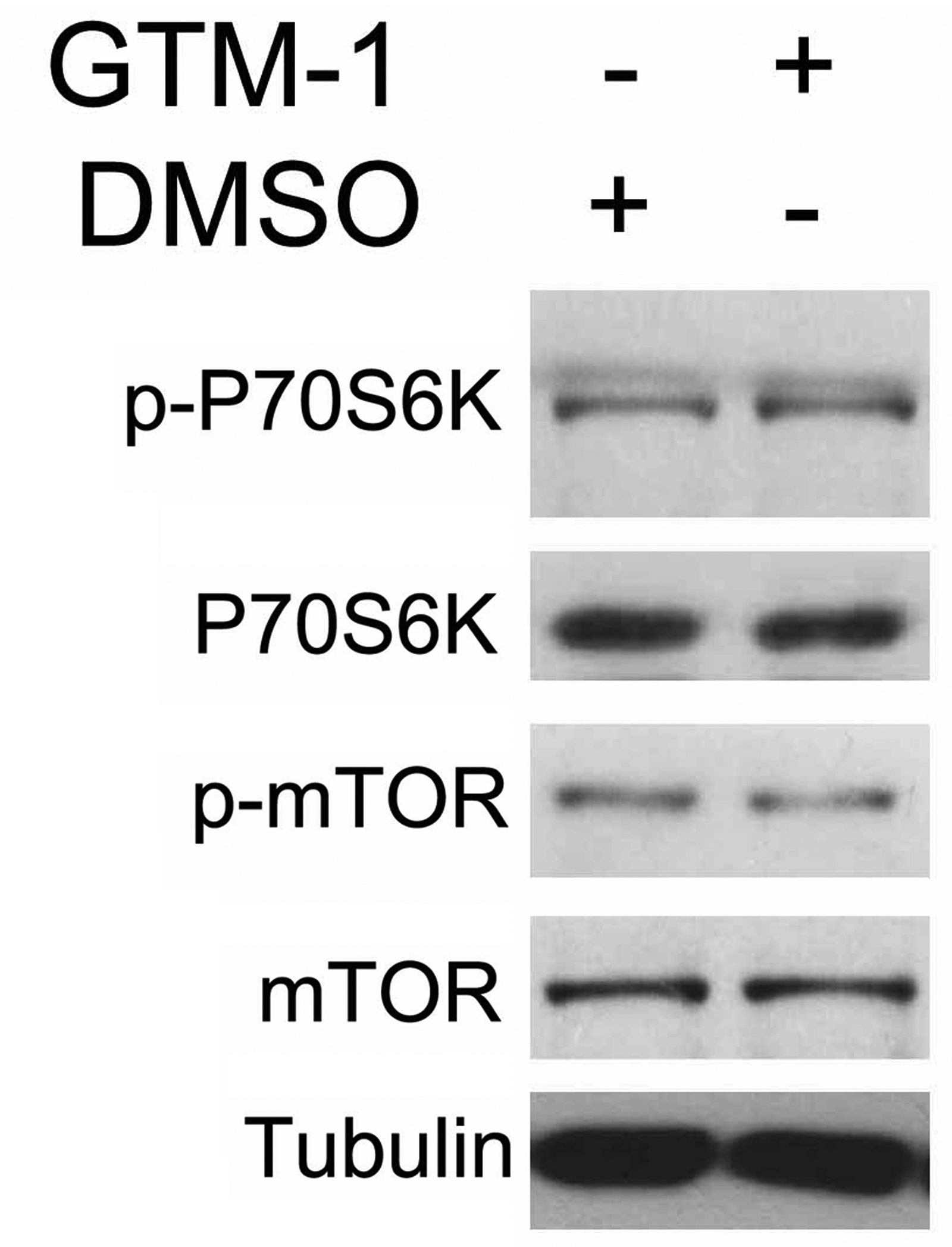

Supplement: Figure S4 — Five-month-old 3XTg-AD mice were administered daily with GTM-1 (3 mg/kg/day) or with vehicle (WEH) for 8 weeks. Brain proteins were obtained from 8 different 3xTg-AD mice treated with GTM-1 and 8 different 3xTg-AD mice treated with vehicle after 8 weeks of treatment, and the protein levels or protein phosphorylation levels were assessed with the indicated antibodies. (TIF) [file pone.0065367.s004.tif]
